# Factors associated with COVID-19 vaccination among pregnant women in Rio De Janeiro City, Brazil

**DOI:** 10.1038/s41598-023-44370-6

**Published:** 2023-10-25

**Authors:** Moara Alves Santa Bárbara Borges, Pilar Tavares Veras Florentino, Thiago Cerqueira-Silva, Luciana Freire de Carvalho, Vinícius de Araújo Oliveira, Gislani Mateus Oliveira Aguilar, Rodrigo de Sousa Prado, Daniel Soranz, Guilherme Loureiro Werneck, Julia M. Pescarini, Paulo Sérgio Sucasas da Costa, Mauricio Lima Barreto, Márcio Henrique de Oliveira Garcia, Gerson Oliveira Penna, Manoel Barral-Netto, Enny S. Paixão

**Affiliations:** 1 Instituto de Patologia Tropical e Saúde Pública da Universidade Federal de Goiás, Goiânia, 74605-050 Brazil; 2 Programa de Pós-Graduação em Ciências da Saúde da Faculdade de Medicina da Universidade Federal de Goiás, Goiânia, 74605-050 Brazil; 3 Centro de Integração de Dados e Conhecimento para Saúde (CIDACS), Instituto Gonçalo Moniz, Fundação Oswaldo Cruz, Salvador, 40296-710 Brazil; 4 Secretaria Municipal de Saúde, Rio de Janeiro, 20211-110 Brazil; 5 Departamento de Epidemiologia, Instituto de Medicina Social, Universidade do Estado do Rio de Janeiro, Rio de Janeiro, 20550-013 Brazil; 6 Instituto de Estudos em Saúde Coletiva, Universidade Federal do Rio de Janeiro, Rio de Janeiro, 21941-592 Brazil; 7 Department of Infectious Diseases Epidemiology, London School of Hygiene and Tropical Medicine, London, WC1E 7HT UK; 8 Secretaria de Vigilância em Saúde e Ambiente, Ministério da Saúde, Brasília, 70723-040 Brazil; 9 Núcleo de Medicina Tropical, Universidade de Brasília, Escola de Governo Fiocruz Brasília, Brasília, 70904-130 Brazil; 10 Faculdade de Medicina, Universidade Federal da Bahia, Salvador, 40110-100 Brazil

**Keywords:** Vaccines, Health policy, Epidemiology, Viral infection

## Abstract

COVID-19 vaccination during pregnancy is safe and effective in reducing the risk of complications. However, the uptake is still below targets worldwide. This study aimed to explore the factors associated with COVID-19 vaccination uptake among pregnant women since data on this topic is scarce in low-to-middle-income countries. A retrospective cohort study included linked data on COVID-19 vaccination and pregnant women who delivered a singleton live birth from August 1, 2021, to July 31, 2022, in Rio de Janeiro City, Brazil. Multiple logistic regression was performed to identify factors associated with vaccination during pregnancy, applying a hierarchical model and describing odds ratio with 95% confidence intervals. Of 65,304 pregnant women included in the study, 53.0% (95% CI, 52–53%) received at least one dose of COVID-19 vaccine during pregnancy. Higher uptake was observed among women aged older than 34 (aOR 1.21, 95%CI 1.15–1.28), black (aOR 1.10, 1.04–1.16), or parda/brown skin colour (aOR 1.05, 1.01–1.09), with less than eight years of education (aOR 1.09, 1.02–1.17), living without a partner (aOR 2.24, 2.16–2.34), more than six antenatal care appointments (aOR 1.92, 1.75–2.09), and having a previous child loss (OR 1.06, 1.02–1.11). These results highlight the need for targeted educational campaigns, trustful communication, and accessibility strategies for specific populations to improve vaccination uptake during pregnancy.

## Introduction

COVID-19 during pregnancy and puerperium is associated with higher morbidity and mortality risk, including hospitalisation, use of respiratory support, and admission to an intensive care unit when compared to non-pregnant pairs^1–3^. In 2021 in Brazil, for example, the estimated COVID-19 case fatality rate among pregnant and puerperal women was 7.2%, compared to 2.8% in the general population^4^. Furthermore, COVID-19 has been linked to adverse perinatal events, such as preterm birth, fetal loss, and neonatal mortality^2, 5^.

COVID-19 vaccination in pregnant women is a safe and effective preventive measure that can reduce the risk of COVID–19–associated complications for both mother and child^6–11^. However, COVID-19 uptake is still below targets worldwide. According to a systematic review, in 2021, the average uptake by the time of delivery was 27.5%^12^, reaching 78% in Sweden and 87% in Norway^13^. In addition, most studies evaluating COVID-19 vaccination among pregnant women identified older age, white self-identified skin colour, high level of education, gravidity, and high socioeconomic status as factors associated with vaccination, predominantly in high-income countries^9, 12–14^.

**Figure 1:**
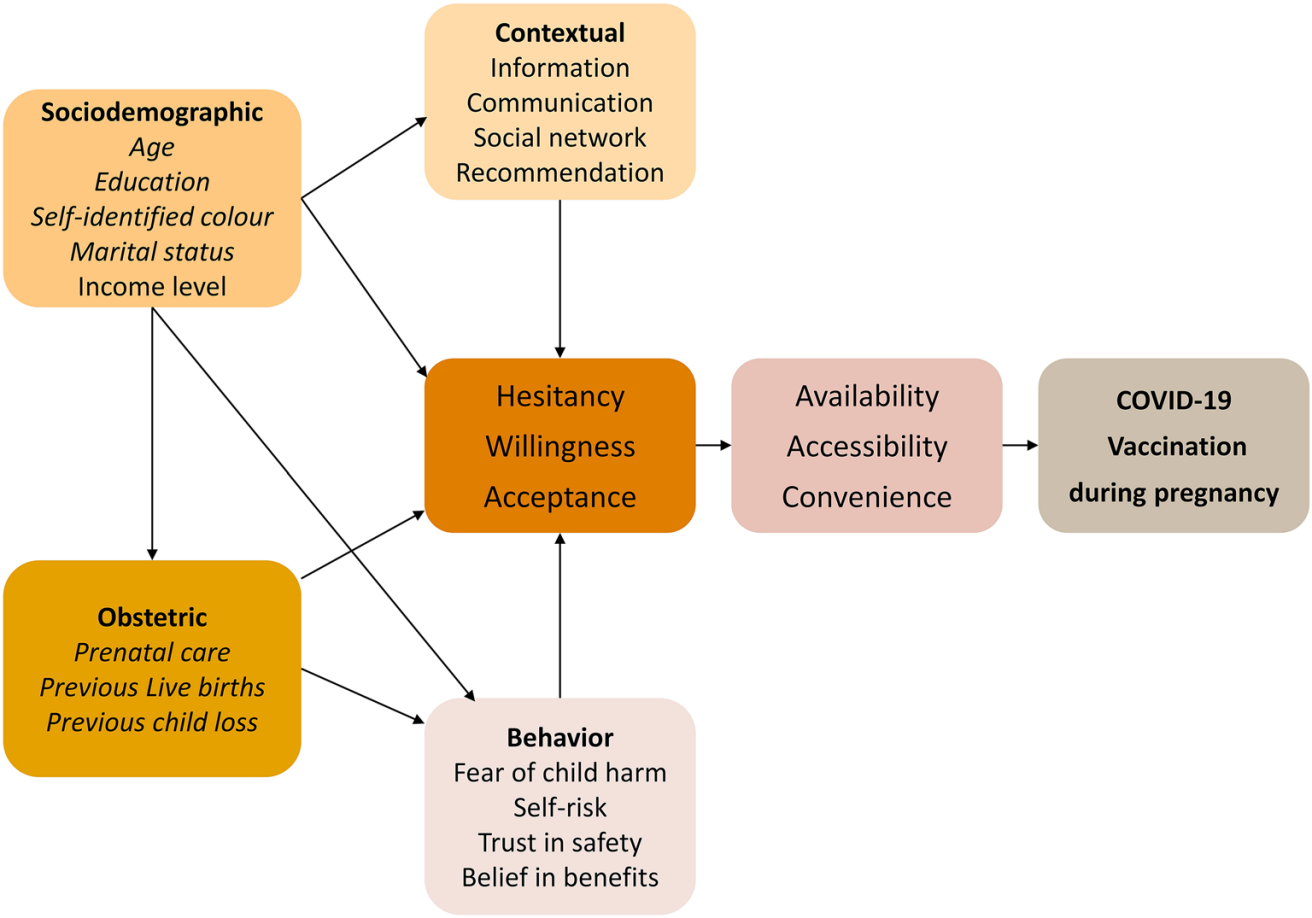
A conceptual framework describing factors that can contribute to COVID-19 vaccine uptake during pregnancy. The variables highlighted were included for analysis in this study.

Based on the literature, we developed a conceptual framework describing the potential factors associated with COVID-19 vaccination during pregnancy (Fig. 1)^9, 12, 15–19^. Sociodemographic aspects, obstetric history, social context, beliefs, and behaviours can contribute to the mother's acceptance and vaccination uptake and therefore were included in the conceptual framework.

Identifying factors associated with COVID-19 vaccine uptake in pregnant women is relevant to support public health decision-making and improve vaccine equity and coverage while addressing regional health disparities related to age, skin colour, education level, income, and access to antenatal care. However, these data on associated factors, especially from low-middle-income countries, are still scarce. Therefore, this study investigates the sociodemographic and obstetric factors that can contribute to COVID-19 vaccine uptake during pregnancy in Brazil.

## Results

### Study population

**Figure 2:**
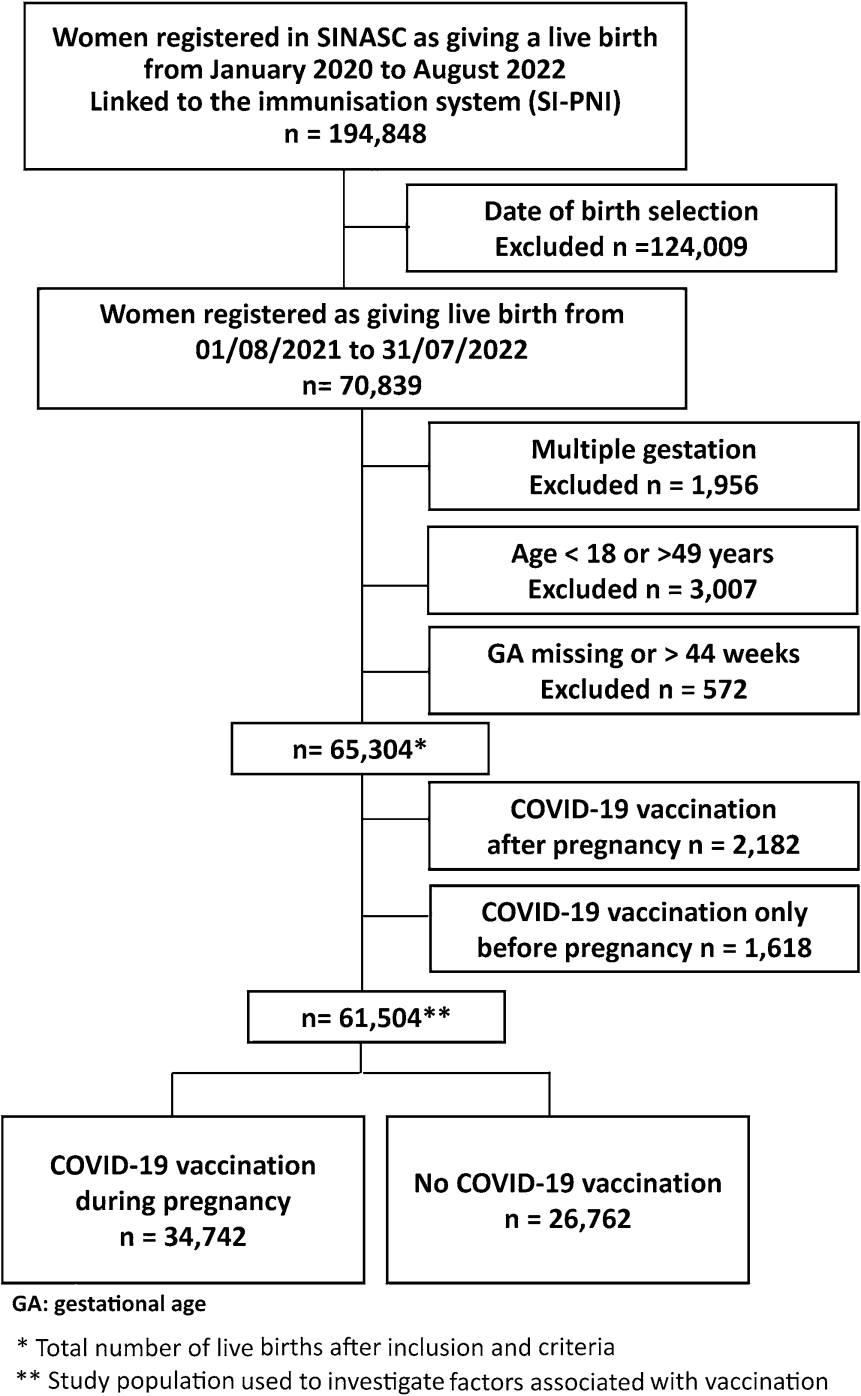
Flowchart demonstrating the study population selection.

**Figure 3:**
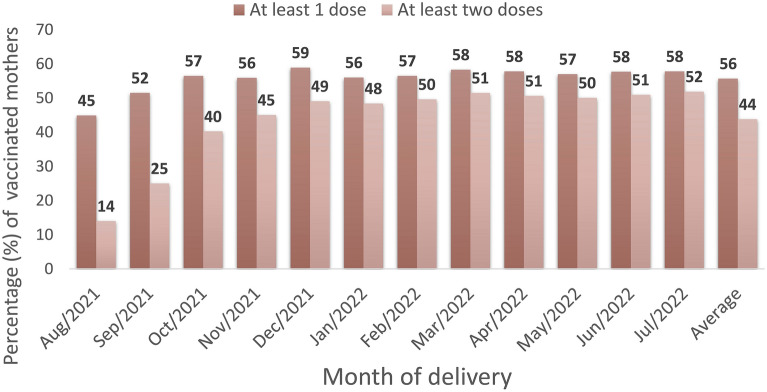
COVID-19 maternal vaccination by month of delivery and number of doses.

During the study period, 65,304 singleton live births from women aged 18–49 years, with gestational age between 22 and 44 weeks, were registered in Rio de Janeiro city. The schematic diagram describes our data selection process and the study population (Fig. 2; Supplementary Figure 1 and Table 3). About 56% (95% CI, 55–56%) of our study population received at least one dose of the COVID-19 vaccine, and 44% (95% CI, 43–44%) at least two doses up to delivery, including those vaccinated before and during pregnancy (Fig. 3; Supplementary Table 4). The COVID-19 vaccine uptake during pregnancy, with at least one dose, was 53% (34,742/65,304; 95% CI, 52-53%) (Supplementary Table 3). Of those mothers vaccinated during pregnancy, the platforms most used were BNT162b2 (Pfizer/Biotech) (68%) and CoronaVac (Sinovac/Butantan) (27%) in at least one dose, mainly during the second and third trimesters of pregnancy (Supplementary Tables 5 and 6).

Table 1 describes the characteristics of those vaccinated before pregnancy, during pregnancy, and those not vaccinated during the study period. Women vaccinated only before pregnancy differed slightly from those vaccinated during pregnancy (Table 1). They had higher proportions of age > 34 years (34% × 22%), at least 12 years of education (45% × 28%), self-identified white skin colour (40% × 32%), living with a partner (42% × 28%), and a higher proportion of a previous child loss (26% × 23%). Most of the women in the three groups started antenatal care in the first trimester and had more than six visits. Women who were never vaccinated, when compared with those vaccinated during pregnancy, were more likely to have 12 years or more of education (37%× 28%), self-identified as white (38% × 32%), and live with a partner (47% × 29%) (Table 1).

**Table 1:** Sociodemographic and obstetric characteristics by vaccination status.

	Never Vaccinated	Vaccinated up to delivery	
	No COVID-19 vaccine	COVID-19 vaccine before pregnancy	COVID-19 vaccine during pregnancy
	*n* = 26,762	*n* = 1,628	*n* = 34,742
Sociodemographic	n (%)	n (%)	n (%)
Age (years) – median, IQR	29 (24–34)	31 (26–36)	29 (23–34)
18–24	7,197 (26.9)	338 (20.8)	11,252 (32.4)
25–34	13,468 (50.3)	740 (45.5)	15,852 (45.6)
≥ 35	6,097 (22.8)	550 (33.8)	7,638 (22.0)
Education (years)			
0–7	2,685 (10.3)	138 (8.6)	4,216 (12.5)
8–11	13,796 (52.9)	737 (46.1)	20,037 (59.5)
≥ 12	9,578 (36.8)	722 (45.2)	9,400 (27.9)
Self-identified skin color			
White	9,726 (38.2)	600 (39.7)	10,836 (32.5)
Black	3,746 (14.7)	231 (15.3)	5,609 (16.8)
Parda / Brown	11,788 (46.3)	666 (44)	16,689 (50)
Asian	195 (0.8)	13 (0.9)	187 (0.6)
Indigenous	28 (0.1)	3 (0.2)	38 (0.1)
Marital status			
Without a partner	13,976 (52.7)	927 (57.5)	24,567 (71.3)
With a partner	12,562 (47.3)	684 (42.5)	9,869 (28.7)
Obstetric			
Beginning of antenatal care			
First trimester	20,544 (86.5)	1,276 (89.0)	27,390 (88.3)
Second trimester	2,768 (11.7)	132 (9.2)	3,195 (10.3)
Third trimester	426 (1.8)	26 (1.8)	445 (1.4)
Antenatal care appointments			
0 to 3	1,374 (5.4)	86 (5.7)	1,190 (3.6)
4 to 6	3,699 (14.6)	186 (12.3)	4,272 (12.8)
> 6	20,301 (80.0)	1,238 (82.0)	27,922 (83.6)
Gestational age at birth (weeks)	38 (38–40)	38 (37–39)	38 (38–40)
Previous gestation			
Nulliparous (0)	10,019 (38.0)	535 (33.9)	12,775 (37.4)
1 to 2	12,738 (48.3)	810 (51.3)	16,206 (47.4)
Multiparous (≥ 3)	3,628 (13.8)	233 (14.8)	5,222 (15.3)
Previous live births			
0	11,913 (45.3)	666 (42.4)	15,259 (44.8)
1 to 2	12,227 (46.4)	772 (49.2)	15,669 (46)
≥ 3	2,184 (8.3)	132 (8.4)	3,170 (9.3)
Previous child loss			
None	20,379 (78.2)	1,108 (74.1)	25,905 (76.9)
One or more	5,675 (21.8)	387 (25.9)	7,786 (23.1)

IQR: interquartile range.

Missing values not included in proportions: education (*n* = 1,792); self-identified skin colour (*n* = 2,662); marital status (*n* = 530); beginning of antenatal care (*n* = 6,736); number of antenatal care appointments (*n* = 2,746); previous gestation (*n* = 916); number of previous live births (*n* = 1,082); number of a previous child loss (*n* = 1,759).

### Factors associated with vaccine uptake

**Figure 4:**
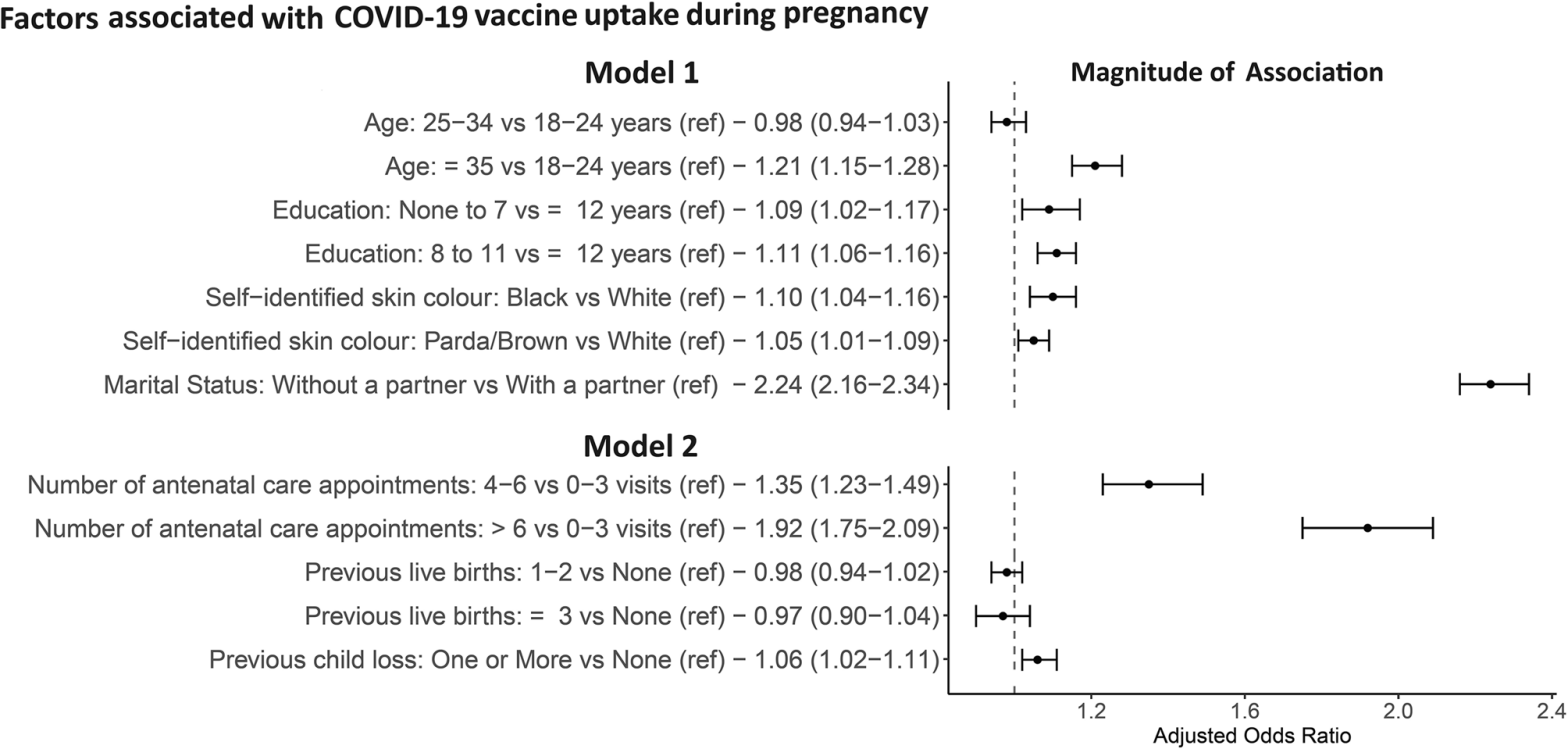
Forest plot of the magnitude of association between the factors associated with the uptake of the COVID-19 vaccine during pregnancy. Ref: variable used as the reference in the multiple logistic regression. Model 1: included socioeconomic (age, education, self-identified skin colour, marital status) Model 2: included obstetric (the total number of antenatal care appointments, the number of previous live births, the number of previous child loss) and socioeconomic.Month-year of birth and the gestational week at delivery are included as confounding variables in both models.

In an unadjusted analysis, age, education level, self-identified skin colour, antenatal care, number of live births, and previous child loss were potentially associated with COVID-19 vaccination during pregnancy (Supplementary Table 7). In the first model of the multiple logistic regression (including the block of socioeconomic variables and confounders factors), women older than 34 years (aOR 1.21, 95% CI 1.15–1.28) were more likely to be vaccinated than those younger than 25. In addition, women living without a partner (aOR 2.24, 95% CI 2.16–2.34), of black (aOR 1.10, 95% CI 1.04–1.16) or parda/brown (aOR 1.05, 95% CI, 1.01–1.09) self-identified skin colour, with a low level of education (0-7 years aOR 1.09, 95% CI 1.02–1.17; 8–11 years aOR 1.11, 95% CI, 1.06–1.16) were more likely to be vaccinated than white women, with 12 years or more of education, living with a partner (Fig. 4 and Supplementary Table 7).

In the second model (including obstetric variables in the presence of socioeconomic variables and confounders), the likelihood of vaccination increased progressively with the number of antenatal care appointments compared to less than 4 (4-6 aOR 1.35, 95% CI 1.23-1.49; > 6 aOR 1.92, 95% CI 1.75-2.09). Having at least a previous child loss (aOR 1.06, CI 95% 1.02-1.11) was a factor potentially associated with vaccination during pregnancy, compared to none. The number of previous live births was not associated with uptake among pregnant women (Fig. 4 and Supplementary Table 7).

## Discussion

This study estimated COVID-19 vaccine uptake during pregnancy as 53%, with most pregnant women receiving Pfizer and CoronaVac vaccines. The likelihood of a COVID-19 vaccine uptake was higher among women of older age, living without a partner, and with more antenatal care appointments. With lower magnitude, self-identify as black and brown skin colour, low educational level, and a previous child loss were also associated with vaccine uptake in this population.

Despite the well-defined maternal and neonatal COVID-19 risks and the vaccine's proven benefits, the vaccine uptake among pregnant women was lower than among the general population, which was 89%. Yet, it was similar to or higher than the COVID-19 vaccine uptake during pregnancy in different countries, for example, the United Kingdom (32–66%), the United States (16–22%), Palestine (25%), and Mexico (20%)^8, 14, 20–24^.

In the cohort study presented herein, women older than 34 had a 21% higher chance of being vaccinated than those younger than 25, consistent with previous studies^12, 25, 26^. We also found higher odds of vaccination among pregnant women with less than 12 years of education when compared to less than eight, contrary to the findings of other studies conducted in middle-high-income countries^13, 15, 16, 20, 24, 27^ but similar to a Chinese survey that observed low acceptance of COVID-19 vaccination in women with a high level of education^25^. The authors hypothesise that this group could be more concerned about safety and side effects^25^.

In our population, black and parda/brown skin colour were associated with COVID-19 vaccine uptake during pregnancy; however, the majority of studies pointed to black, non-white and Hispanic races as less likely to be vaccinated than white ^13, 15, 16, 20, 24, 26, 27^. These conflicting findings on self-identified skin colour and education could be related to Brazilian social inequities, regional diversity, the political context, and the accessibility to vaccines only available in the public health system. This enables the access of pregnant women to vaccination while they attend public antenatal care, probably receiving the recommendation and the shot at the same visit. Historically, the Unified Public Health system assists proportionally more people from lower income levels, from black and brown self-identified colour, and with low educational levels ^28^; this could explain higher uptake in these groups.

Pregnant women without a partner were more than twice as likely to be vaccinated than those living with a partner in our study. This contrasts with findings among women of reproductive age in the US, for which being single was associated with 29% lower COVID-19 vaccination likelihood ^29^. Moreover, single mothers from low-middle-income countries are more frequently the economic providers of the family. Thus, their own decision to vaccinate to prevent getting ill could be considered a way of avoiding absenteeism and maintaining their work capacity and employment during the COVID-19 pandemic^21, 30^.

Previous studies showed that more antenatal care appointments, prenatal nurse follow-ups, and being counselled about vaccination by a health care provider increase the likelihood of vaccination during pregnancy. Conversely, antenatal care onset after the first quarter of pregnancy decreases the uptake^31–33^. These findings demonstrate the impact of early and complete antenatal care as an essential measure for vaccine education, recommendation, and accessibility during pregnancy. Rio de Janeiro's well-established maternal health program maintains more than 80% of women on early and complete antenatal care. Additionally, a history of miscarriage may influence the perception of COVID-19 susceptibility, severity, and vaccine benefits^21, 32^. Therefore, women with a history of fetal loss could have been more preventive during their pregnancy, hence, accepting the vaccination as a protection for themselves and the unborn child.

To note, we also described the subset of women vaccinated only before pregnancy as having a higher proportion of older age, white self-identified skin colour, and more than 12 years of education; however, this group wasn’t the objective of this study. It is possible that these women were prioritised to receive the COVID-19 vaccine earlier due to pre-existing conditions, high risk of complications, or increased exposure as healthcare workers.

The high quality of the Brazilian data, the population size and the breadth of data are strengths of this study. Furthermore, it is one of the first studies to address factors associated with COVID-19 vaccine uptake in pregnant women in Brazil and provide new evidence of low vaccine uptake in this population.

This study has some limitations, particularly related to the use of secondary data sources. Routinely administrative data are not collected for the research proposal and have limited information available. Therefore, we were not able to study other important variables, such use of private insurance. Linkage error is another potential limitation. Although linkage errors can occur, and some women may be more likely to link than others, the linkage approach was bespoke, and a manual review was performed to improve linkage accuracy. Additionally, it is important to note that incomplete data can occur. However, the amount of missing data for each variable was relatively low (Supplementary Table 8).

Our study did not include pregnant women who had an abortion, a stillbirth, or multiple gestations; therefore, the results presented in this study cannot be generalized to these groups. Wide misinformation released on social media, concerns regarding vaccine safety, scepticism about the disease risk and the political context could have influenced the uptake among pregnant women. Yet, they were not addressed herein and should be better investigated in further studies^15, 34–36^. Moreover, factors associated with vaccine uptake may vary from one place to another; therefore, it cannot be generalised to other Brazilian municipalities.

In conclusion, COVID-19 vaccination uptake remains poor among pregnant women in Brazil. There is a higher uptake among those older than 34 years, of black or brown self-identified skin colour, with a low level of education, living without a partner, having more than six appointments, and with a previous child loss in Rio de Janeiro city. These findings contribute to planning targeted educational campaigns, trustful communication, and accessibility strategies to specific populations to improve vaccine uptake since pregnant women widely benefit from COVID-19 vaccination. Factors associated with vaccination likely vary from one place to another and by population; therefore, further local studies are needed to guide intervention and address the impact of socioeconomic, socio-political, and social media on immunisation uptake.

## Methods

### Study setting, study design, and population

This retrospective cohort study used linked data from the live birth and vaccination information systems in Rio de Janeiro city. Rio de Janeiro is the second largest city in Brazil, with an estimated population of 6,700,000. It has a monthly per capita income of 4 minimum wage, considered a high-income city compared to other regions of the country^37^ In 2021, the number of live births was 67,973^38^. In July 2022, the coverage of COVID-19 vaccination was 89% for the complete primary regimen and 57% for a booster dose in the general population above 18 years^39^.

The COVID-19 immunisation started in Rio de Janeiro city on January 20, 2021^40^, initially for healthcare professionals, adults older than 60 years, and high-risk groups. Pregnant women with comorbidities started vaccination in March 2021; by May 2021, it was interrupted due to a severe adverse event related to the COVID-19 vaccine in this population. From July 07, 2021, vaccination was resumed and made available for all pregnant, breastfeeding or women planning to become pregnant^41^. CoronaVac (Sinovac/Butantan) and BNT162b2 (Pfizer/Biotech) were the recommended platforms.

For this investigation, we included all women who delivered live births in Rio de Janeiro city between 1st August 2021 and 31 July 2022. Our exclusion criteria were multiple births, ages less than 18 years or higher than 49 years, and records with missing or implausible gestational ages (> 44 weeks) (Fig. 2).

### Data sources and linkage process

The Declaration of Live Birth, a legal document filled out by the health care professional who attends the birth, is entered into the Live Birth Information System (SINASC). It contains details on the mother (such as age, education, skin colour, and marital status), about the pregnancy (such as antenatal appointments, the gestational period, prior gestations, previous live births, and previous losses), and details about the newborn (e.g., birth weight, sex, APGAR score). In addition, all vaccinations provided in Brazil are documented in the National Immunization Program Information System (SI-PNI), along with the administration date of the first, second, and booster doses, with its platform type. The SINASC data initially available had records from women who gave a live birth in Rio de Janeiro city from January 1, 2020, to August 28, 2022. The SI-PNI data included vaccination records from January 19, 2021, to August 31, 2022 (Supplementary Figure 1, Table 1 and Table 2). The linkage between records from SI-PNI and SINASC allowed access to any vaccination that happened before, during and after the pregnancy period.

The matching process used the maternal name, date of birth, zip code, and neighbourhood. We used the Jaro-Winkler string comparator to compare the similarity between string variables recorded in SINASC and SI-PNI. This algorithm calculates the similarity between two strings based on the number of shared characters and transpositions^42^. The resulting similarity score ranges between 0 (no similarity) and 1 (perfect similarity). We categorised the similarity scores into three categories: (0, 0.85), (0.85, 0.95), and (0.95, 1)]. We employed a three-step approach that checked for a string similarity score greater than 0.95, followed by exact matches for dates of birth and zip code. Any potential matches then underwent a manual review. After data linkage, the individual identifiers were removed, and the de-identified dataset was made available for analysis.

### COVID-19 vaccine status

We estimated the date of the last period (DLP) using the date of delivery minus the days of pregnancy according to the gestational age at birth. We determined the date of conception by adding 14 days to the DLP. We defined the gestational period as the time between the date of conception and the date of birth. The vaccination status was determined using the dates of the vaccination registries compared to the gestational period.

Women who received at least one dose between the conception date and the date of delivery were considered vaccinated during pregnancy. Those who received all registered doses before the pregnancy period (before the conception date) were grouped as vaccinated before pregnancy. Those who received vaccines exclusively after the delivery date were assigned as vaccinated after pregnancy. Finally, women with no register of a vaccine dose were regarded as never vaccinated. We estimated vaccine uptake during pregnancy as the proportion of women who received any vaccination during pregnancy as a percentage of all births (Supplementary Table 3).

### Variables

We divided variables into sociodemographic: age (18–24, 25–34, ≥35 years), education (0–7, 8–11, ≥12 years), self-identified skin colour (black, parda/brown, white, Asian, or Indigenous), and marital status (with or without a partner). And obstetric: the trimester of the first antenatal care appointment (first, second, or third), the total number of appointments (0–3, 4–6, or >6), the number of previous gestations (none, 1–2, or ≥3), the number of previous live births (none, 1–2, or ≥3), and the number of previous child loss (none or at least one). The Indigenous and Asian races were presented in the descriptive analysis and excluded from the logistic regression due to the small sample size.

We considered the length of pregnancy and the burden of COVID-19 infection during the study period to be a confounder a priori. Therefore, we included the month-year of birth and the gestational week at delivery as additional variables in the analyses.

### Statistical analysis

We assessed each group’s characteristics by describing categorical variables as frequencies and percentages, excluding missing data. Continuous variables, such as age and gestational age, were presented as the median and interquartile range (IQR). In the descriptive analysis, we stratified the groups by being vaccinated only before pregnancy, vaccinated with at least one dose during pregnancy, and never vaccinated.

To identify the factors associated with vaccine uptake in pregnant women, we compared only the population of women vaccinated during pregnancy with those who were never vaccinated during the study period. For each potential factor associated with uptake, we ran a bivariate logistic regression individually, describing the crude odds ratio (OR) and its associated 95% confidence interval, controlled by gestational age and month-year of birth (Supplementary Table 7).

In addition, we performed a multiple logistic regression using a hierarchical framework with two levels. In the first level, we had the socioeconomic variables: age, education, self-identified skin colour, and marital status. In the second level, we included all the variables above and the obstetrics variables: the total number of antenatal appointments, the number of previous live births, and the number of previous child losses.

In the first model of multiple logistic regression, we included only the socioeconomic variables. The overall effect of socioeconomic factors (the distal factors) was assessed in this model 1. In the second model, we included the obstetric variables in addition to the sociodemographic block. Therefore, the unconfounded effect of the obstetric variables was obtained in this model. Both models were also controlled by gestational age and month-year of birth. Missing data on each covariate were excluded from the analysis (Supplementary Table 8). The final adjusted odds ratio and 95% confidence intervals were described for each model separately (Supplementary Table 7). Data management and statistical analysis were conducted using IBM SPSS, Statistical Package for the Social Sciences, Version 28.0 (Armonk, NY: IBM Corp). The de-identified dataset and the programming codes can be made available under the request.

### Ethics

The present study has been approved by the Ethics Committee of Research Center Gonçalo Moniz /Oswaldo Cruz Foundation, Salvador, Bahia, Brazil (IORG 0002090/OMB No. 0990-0279 valid until 01/27/2025), under the Certificate of Submission for Ethics Review No 63287822.0.0000.0040. The protocol and procedures presented in the project are in full accordance with the Brazilian legislation (Resolution CNS 466/2012) and the declaration of Helsinki regarding ethical standards in conducting research involving human beings. Due to the retrospective nature of the study, the need for informed consent was waived by the Ethics Committee of Research Center Gonçalo Moniz /FIOCRUZ/BA.

### Supplementary Information

Supplementary Information.

## Data Availability

All raw data from the information systems are available at: http://sistemas.saude.rj.gov.br/tabnet/tabcgi.exe?sinasc/nascido.def -http://sistemas.saude.rj.gov.br/tabnetbd/dhx.exe?pni_covid/pni_covid.def. The de-identified linkage dataset and the programming codes could be made available under the request by contacting the corresponding author MASBB.
