# Supplementary material for: Factors associated with COVID-19 vaccination among pregnant women in Rio De Janeiro City, Brazil
Source: Sci Rep. 2023 Oct 25;13:18235. doi: 10.1038/s41598-023-44370-6 (PMC10600223; doi:10.1038/s41598-023-44370-6)
Supplement: Supplementary file 1 — Supplementary Information. [file 41598_2023_44370_MOESM1_ESM.pdf]

Factors associated with COVID-19 vaccination among pregnant women in Rio de Janeiro City, Brazil

SUPPLEMENTARY MATERIAL

All raw data from the information systems are available at:

- http://sistemas.saude.rj.gov.br/tabnet/tabcgi.exe?sinasc/nascido.def
- http://sistemas.saude.rj.gov.br/tabnetbd/dhx.exe?pni_covid/pni_covid.def

The de-identified linkage dataset and the programming codes could be made available under the request.

1. **Dataset information before linkage**

Table 1. The date range of live birth records from the **Live Birth Information System (SINASC) before linkage and inclusion and exclusion criteria**

**Date of birth**		
N	Valid	194848
	Missing	0
Minimum		01-JAN-2020
Maximum		28-AUG-2022

Table 2. The date range of vaccination records from the National Immunization Program Information System (SI-PNI) before linkage

Date of vaccine doses		Date of first	Date of second	Date of Jansen's	Date of third	Date of fourth
N	Valid	108875	99563	3933	60618	13805
	Missing	85973	95285	190915	134230	181043
Minimum		19-JAN-2021	19-JAN-2021	19-JAN-2021	22-JUN-2021	05-FEB-2021
Maximum		23-AUG-2022	31-AUG-2022	31-AUG-2022	22-JUL-2022	31-AUG-2022

2. **Study population selection after the linkage**

2.1 Inclusion criteria

Date of birth 01/08/21 - 31/07/22
Age 18 ≥ Age ≤49
Complete data on Gestational Age (GA)

2.2 Exclusion criteria

Implausible GA (>44 weeks)
Multiple births

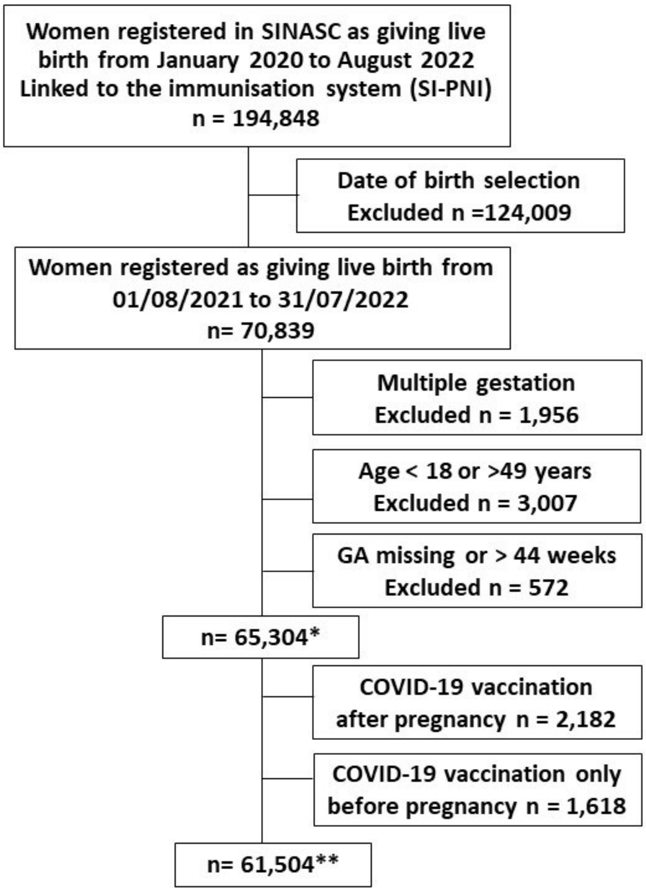

Figure 1. Flow chart of study population selection

3. **Population after inclusion and exclusion criteria (Figure 2)**

Table 3. Population groups by vaccination

**Groups**	**Frequency (n)**	**Percentage (%)**
**Never vaccinated**	26,762	41.0
**Vaccinated during pregnancy**	34,742	53.0
**Vaccinated before**	1,628	2.6
**Vaccinated after**	2,172	3.4
**Total**	65,304	100.0

4. **Maternal Vaccination up to delivery (Figure 3)**

Table 4. The proportion of COVID-19 Maternal Vaccination (Figure 3)

**Month of delivery**	**Total births**	**At least 1 dose (n)**	**At least 1 dose (%)**	**At least two doses (n)**	**At least two doses (%)**
**Aug/2021**	5,566	2,499	45	778	14
**Sep/2021**	5,507	2,836	52	1,377	25
**Oct/2021**	5,300	2,992	57	2,131	40

**Nov/2021**	5,422	3,030	56	2,441	45
**Dec/2021**	5,457	3,213	59	2,678	49
**Jan/2022**	5,562	3,114	56	2,691	48
**Feb/2022**	5,426	3,068	57	2,692	50
**Mar/2022**	6,017	3,506	58	3,098	51
**Apr/2022**	5,642	3,262	58	2,856	51
**May/2022**	5,783	3,296	57	2,895	50
**Jun/2022**	5,169	2,981	58	2,633	51
**Jul/2022**	4,453	2,573	58	2,310	52
**Average**	65,304	36,370	**56**	28,580	**44**

5. **Vaccination during Pregnancy**

Table 5. Type of vaccines and doses received during pregnancy

	**d1**		**d2**		**d3**		**d4**			
	n	%	n	%	n	%	n	%	Total	%
**AstraZeneca**	1,472	5.7	962	3,7	180	2,2	3	2,7	2,617	4.4
**Coronavac**	7,910	30.4	7,618	29,6	592	7,3	25	22,7	16,145	**27.0**
**Pfizer**	16,518	62.5	17,137	66,6	7250	89,0	80	72,7	40,985	**68.3**
**Janssen**	105	0.0			128	1,6	2	1,8	235	0.3
**Total**	26,005	100,0	25,717	100,0	8150	100,0	110	100,0	59,982	100

Table 6. Trimester of vaccination by type of dose

**Trimester**	**d1**	**d2**	**d3**	**d4**	**total**
**First (0-97 days)**	10	7,299	480	1	7,790
**Second (98-195 days)**	10,856	10,441	3,423	5	**24,725**
**Third (>195 days)**	4,892	7,977	4,247	104	**17,220**
**Total**	26,005	25,717	8,150	110	59,982

6. **Results of bivariate regression and models of multivariate logistic regression**

Table 7. Association of COVID-19 vaccination uptake during pregnancy compared to no vaccination

**Determinants**	**Bivariate logistic regression**	**Multiple logistic regression**
	OR (CI 95%)	aOR (CI 95%)
**Sociodemographic**		**Model 1[a]**
**Age (years)**		
18-24 (ref)	1	1
25-34	0.75 (0.73-0.78)	0.98 (0.94-1.03)
≥35	0.81 (0.77-0.85)	1.21 (1.15-1.28)
**Education (years)**		
None to 7	1.60 (1.51-1.70)	1.09 (1.02-1.17)
8 to 11	1.48 (1.43-1.53)	1.11 (1.06-1.16)
≥ 12 (ref)	1	1

**Self-identified skin colour**		
White (ref)	1	1
Black	1.34 (1.28-1.41)	1.10 (1.04-1.16)
Parda/Brown	1.27 (1.23-1.32)	1.05 (1.01-1.09)
**Marital status**		
Without a partner	2.24 (2.16-2.31)	2.24 (2.16-2.34)
With partner (ref)	1	1
**Obstetric**		**Model 2[b]**
**Antenatal care appointments**		
0-3 (ref)	1	1
4-6	1.33 (1.21-1.45)	1.35 (1.23-1.49)
> 6	1.55 (1.43-1.70)	1.92 (1.75-2.09)
**Previous live births**		
0 (ref)	1	1
1 to 2	1.00 (0.96-1.03)	0.98 (0.94-1.02)
≥3	1.14 (1.07-1.21)	0.97 (0.90-1.04)
**Previous child loss**		
None (ref)	1	1
One or more	1.09 (1.05-1.13)	1.06 (1.02-1.11)

OR: crude *odds ratio.*
aOR: adjusted *odds ratio.* Hierarchical models 1 and 2 are displayed separately.
Ref: variable used as the reference in regression.
Month-year of conception and the gestational week at delivery are included as confounding variables in both analyses.
[a]Model 1: included sociodemographic (age, education, self-identified skin colour, marital status)
[b]Model 2: included obstetric (the total number of antenatal care appointments, the number of previous live births, the number of previous child loss) and sociodemographic (age, education, self-identified skin colour, marital status)

Table 8. Summary of valid and missing cases by variable

Variables	All cases					
	**Valid cases**		**Missing cases**		**Total**	
	N	%	N	%	N	%
**Age**	61504	100,0%	0	0,0%	61504	100,0%
**Education**	59712	97,1%	1792	2,9%	61504	100,0%
**Self-identified skin colour**	58842	95,7%	2662	4,3%	61504	100,0%
**Marital Status**	60974	99,1%	530	0,9%	61504	100,0%
**Number of antenatal care appointments**	58758	95,5%	2746	4,5%	61504	100,0%
**Previous live births**	60422	98,2%	1082	1,8%	61504	100,0%
**Previous child loss**	59745	97,1%	1759	2,9%	61504	100,0%
